# Integrating post-event very high resolution SAR imagery and machine learning for building-level earthquake damage assessment

**DOI:** 10.1007/s10518-024-01877-1

**Published:** 2024-03-09

**Authors:** Valentina Macchiarulo, Giorgia Giardina, Pietro Milillo, Yasemin D. Aktas, Michael R. Z. Whitworth

**Affiliations:** 1 Department of Geoscience and Engineering, Delft University of Technology, Stevinweg 1, 2628 CN Delft, The Netherlands; 2 Department of Civil and Environmental Engineering, University of Houston, 4226 Martin Luther King Blvd, Houston, 77204 TX USA; 3 Microwaves and Radar Institute, German Aerospace Center (DLR), Münchener Straße 20, 82234 Weßling, Germany; 4 Department of Civil, Environmental and Geomatic Engineering, University College London, Gower Street, London, WC1E 6BT UK; 5 Ground Engineering, AECOM, Tailyour Road, Plymouth, PL6 5DH UK

**Keywords:** Disaster management, Post-earthquake reconnaissance, Remote sensing, Synthetic aperture radar, Texture analysis, ML techniques

## Abstract

Earthquakes have devastating effects on densely urbanised regions, requiring rapid and extensive damage assessment to guide resource allocation and recovery efforts. Traditional damage assessment is time-consuming, resource-intensive, and faces challenges in covering vast affected areas, often limiting timely decision-making. Space-borne synthetic aperture radars (SAR) have gained attention for their all-weather and day-night imaging capabilities. These advantages, coupled with wide coverage, short revisits and very high resolution (VHR), have created opportunities for using SAR data in disaster response. However, most SAR studies for post-earthquake damage assessment rely on change detection methods using pre-event SAR images, which are often unavailable in operational scenarios. Limited studies using solely post-event SAR data primarily concentrate on city-block-level damage assessment, thus not fully exploiting the VHR SAR potential. This paper presents a novel method integrating solely post-event VHR SAR imagery and machine learning (ML) for regional-scale post-earthquake damage assessment at the individual building-level. We first used supervised learning on case-specific datasets, and then introduced a combined learning approach, incorporating inventories from multiple case studies to assess generalisation. Finally, the ML model was tested on unseen study areas, to evaluate its flexibility in unfamiliar contexts. The method was implemented using datasets collected during the Earthquake Engineering Field Investigation Team (EEFIT) reconnaissance missions following the 2021 Nippes earthquake and the 2023 Kahramanmaraş earthquake sequence. The results demonstrate the method’s ability to classify standing and collapsed buildings, achieving up to 72% overall accuracy on unseen regions. The proposed method has potential for future disaster assessments, thereby contributing to more effective earthquake management strategies.

**Supplementary Information:**

The online version contains supplementary material available at 10.1007/s10518-024-01877-1.

## Introduction

Earthquakes have a profound impact on the built environment, resulting in significant human casualties and extensive property damage. In the 21st century alone, approximately 0.78 million deaths were recorded due to earthquakes, with an average of 32,513 fatalities per year between 2000 and 2023 (Ritchie et al. 2022). The earthquakes that have struck densely populated regions in the 21st century serve as reminders of the potential consequences. For instance, the 2011 Tōhoku earthquake and tsunami resulted in property damage estimated between $235 and $305 billion (Kalantari 2012), the 2008 Sichuan earthquake caused over $123 billion in direct damage (The World Bank 2008), and the recent 2023 Kahramanmaraş earthquake sequence led to $34.2 and $5.1 billion in direct damage in Turkey and Syria, respectively (Gunasekera et al. 2023a, b). In response to such catastrophic events, a rapid damage assessment plays a crucial role in guiding resource allocation and facilitating efficient response and recovery efforts.

Post-earthquake reconnaissance activities are instrumental in gathering critical information about the extent and nature of building damage (Wilkinson et al. 2020; Aktas et al. 2022). Traditional methods of damage assessment rely heavily on manual inspections and surveys conducted in the field, where trained personnel document and analyse structural conditions. Whilst these assessments provide valuable insights into the hazards and associated consequences, they are also time-consuming and resource-intensive. The vast scale of affected areas, logistical concerns, limited availability of trained personnel, and safety issues further compound the challenges of conducting timely post-earthquake damage assessment (Wartman et al. 2020), often causing delays in acquiring crucial information for decision-making. As a result, there is a growing recognition of the need for innovative and technology-driven approaches to rapidly assess building damage following an earthquake (Contreras et al. 2021).

Over the past 20 years, remote sensing techniques have emerged as valuable tools for post-earthquake damage assessment, as evidenced by the increasing number of satellite-based emergency mapping activations (Voigt et al. 2016), such as those conducted by the International Charter ‘Space and Major Disasters’ and the Copernicus Emergency Management Service (EMS) in response to major disasters. Among the available remote sensing technologies, satellite Synthetic Aperture Radar (SAR) has gained attention due to its unique all-weather and day-night imaging capabilities (Ge et al. 2020), which allow overcoming the limitations associated with the more commonly used optical data. SAR imagery can provide large area coverage, allowing for the analysis of extensive affected regions, and its short revisit times, enabled by recent SAR satellites constellations (Milillo et al. 2016; Macchiarulo et al. 2022), can provide availability of timely information shortly after a disaster. Additionally, the availability of open data programs (Castelletti et al. 2022) often allows SAR data to be accessible for free in the aftermath of an earthquake, further facilitating its widespread use in disaster response and recovery operations (Giardina et al. 2023; Palamá et al. 2023). Finally, the combination of short wavelengths, such as X-band ($\sim$3 cm), and the spotlight acquisition mode has further enhanced SAR capabilities, enabling the availability of SAR data with sub-metre resolution (Prats-Iraola et al. 2012; Stringham et al. 2019). This very high resolution (VHR) allows SAR sensors to capture details of individual buildings, opening unprecedented opportunities for its operational use in post-earthquake damage assessment (Giardina et al. 2023; Macchiarulo et al. 2023).

Most studies utilising SAR imagery for post-earthquake damage assessment rely on change detection methods that involve either amplitude correlation, interferometric coherence, or a combination of both techniques (Plank 2014; Ge et al. 2020). These methods typically require at least two or three SAR images, including one or two pre-disaster SAR images ideally acquired shortly before the event, and one post-disaster image collected soon after the earthquake. To implement these methods, one or two secondary images are co-registered to one common primary image that serves as a reference. The amplitude correlation method involves computing the amplitude difference and correlation coefficient from two co-registered pre- and post-event SAR amplitude images (Matsuoka and Yamazaki 2004; Trianni and Gamba 2008; Uprety et al. 2013; Gokon et al. 2015). Alternatively, in the coherence-based method interferograms are generated by cross-multiplying the primary image with the two secondary images, resulting in one pre-event Interferometric SAR (InSAR) pair and one co-event InSAR pair (Ge et al. 2015; Yun et al. 2015; Sharma et al. 2017; Natsuaki et al. 2018). Coherence, which indicates the correlation of the phase information, is then computed for both InSAR pairs. In both cases, the change detection process focuses on identifying changes in amplitude or coherence between corresponding image pairs that are likely associated with earthquake-induced damage. However, the reliance on pre-event SAR imagery can limit the applicability of these methods in operational scenarios. For example, VHR satellite acquisitions in Spotlight mode are typically activated only after a seismic event has occurred, resulting in limited availability of pre-event SAR data. Consequently, change detection methods are often unsuitable for analysing VHR SAR data.

To overcome this limitation, some studies have investigated the use of post-event SAR data only for the rapid assessment of earthquake-induced damage (Ge et al. 2020). Some of these methods utilised visual interpretation of post-event SAR amplitude data to identify structures that were destroyed or partially collapsed (Balz and Liao 2010; Jin et al. 2011). Others explored the relationship between damaged buildings and statistical features, such as texture, extracted from the post-event SAR imagery (Dell’Acqua and Polli 2011; Giardina et al. 2023; Macchiarulo et al. 2023), as well as polarimetric features derived from dual or fully-polarimetric post-event SAR data (Li et al. 2012; Zhao et al. 2013; Shi et al. 2015; Zhai and Huang 2016; Bai et al. 2017). It was found that volume scattering extracted from polarimetric data and high-level of entropy (or low-level of homogeneity) derived from single-polarisation data can be correlated with damaged areas. However, the use of polarimetric data was limited by its low resolution and low sensitivity to damage in buildings that were not aligned with the satellite flight direction (Li et al. 2012). Consequently, these methods were designed for a damage assessment at the city block level rather than at the building level. Similarly, in single-polarisation data, the speckle noise inherent in SAR imagery and the differing behaviour of textural features based on imaging geometry presented challenges in the use of individual features for a building-level assessment. As a result, correlation between individual features and damage was only observed when texture values were averaged at the city block level (Dell’Acqua and Polli 2011; Giardina et al. 2023; Macchiarulo et al. 2023).

To leverage the full potential of post-event VHR SAR imagery for building-level assessment, machine learning (ML) techniques could be used to uncover the hidden information embedded in multiple textural features, capturing the spatial patterns and structural variations that are likely associated with earthquake-induced damage. However, current research in this area is limited, with only a few studies exploring the use of multiple textures extracted from post-event VHR SAR imagery in conjunction with ML techniques (Gong et al. 2016; Wu et al. 2016). Moreover, these studies have often focused on testing small areas and a limited number of buildings, making them context-specific and not fully accounting for the complexity and variability of seismic events.

This paper introduces a novel method that combines ML techniques with textural features derived from post-event VHR SAR imagery to classify seismic-induced damage at the building level over large urbanised regions. Inspired by an object-based analysis approach, the proposed method utilises building footprints obtained from geocoded inventories as image objects. We designed a three-level training approach to build a generalised ML model for classifying standing and collapsed buildings in different post-earthquake scenarios. We initially implemented a supervised learning workflow for each case study, training models on corresponding datasets. Subsequently, we proposed a combined learning approach that incorporates inventories from multiple case studies to improve classification performance. Finally, generalisability was assessed by testing the ML model on a new study area, evaluating its adaptability to unfamiliar contexts where training data may be limited. We implemented this method using datasets collected during two Earthquake Engineering Field Investigation Team (EEFIT) reconnaissance missions conducted after the 2021 Nippes earthquake in Haiti (Whitworth et al. 2022) and the 2023 Kahramanmaraş earthquake sequence in Turkey (Aktas et al. 2023). The diversity in damage patterns, spatial scales, and data sources involved - including field-based and remote-sensing-based building damage data, as well as varying VHR SAR imaging acquisition conditions - provided a comprehensive representation of post-earthquake scenarios. This allowed for testing the flexibility of the proposed method in handling the diverse data characteristics typically encountered in operational scenarios. The developed method has potential for advancing future post-earthquake damage assessments.

## SAR background

Space-borne SAR is an active remote sensing technology that maps wide regions of the Earth’s surface by emitting microwave pulses and capturing resulting backscattered signals. SAR satellites follow near-polar orbits, observing the Earth’s surface from south to north (ascending pass) or north to south (descending pass), depending on flight direction. SAR systems use side-looking imaging geometry, with the sensor oriented sideways (left or right) to the flight direction, also known as azimuth. The satellite viewing direction, called Line Of Sight (LOS) or slant-range, is defined by its inclination, or look angle, relative to the nadir.

In SAR images, each pixel is characterised by a phase and amplitude value. The phase measures the signal propagation distance as it travels from the sensor to the target, limited in the range of 0– 2$\pi$. The amplitude quantifies the backscattered energy sensed by the radar, and depends on a target physical (i.e., shape and roughness) and electrical (i.e., permittivity) characteristics. Typically, different wavelength bands lead to diverse penetration levels. Current space-borne SAR sensors mostly operate within three wavelength bands: L-band (24 cm), C-band (5.6 cm), and X-band (3.1 cm). Radar systems with longer wavelengths enable greater electromagnetic penetration (Moreira et al. 2013).

SAR image resolution is primarily determined by acquisition mode, orbital parameters, and sensor characteristics, including sensor bandwidth and look angle. Shorter sensor bandwidths are associated with higher spatial resolutions, with X-band satellites in Spotlight mode achieving the highest resolutions. In the Spotlight mode, the radar beam images a specific point on the Earth for an extended time. This extended imaging time enhances azimuth resolution by increasing illumination time and synthetic aperture length. Consequently, images exhibit higher detail, enabling detection of distinct features in individual structures.

Due to the SAR satellite side-looking geometry, the appearance of buildings within SAR images is affected by geometric distortions, such as shadowing, foreshortening, layover, and multi-bounce reflections (Hanssen 2001). For an idealised flat-roof building, four distinct types of reflections become recognisable (Balz and Liao 2010; Brunner et al. 2010). These include the layover area, corner reflection, roof area, and shadow area. The dihedral corner reflector, formed at the building wall-ground intersection facing the SAR sensor, yields a strong double-bounce reflection, manifesting as a bright linear region within the SAR image. Additionally, the building roof contributes a weaker reflection, resulting in lower-intensity pixels. Depending on building height, this roof reflection could precede the double bounce, creating a layover effect and generating relatively intense pixels. Lastly, a shadowing effect emerges from portions of the building hidden from radar illumination, causing those areas to appear dark in the radar image.

In scenarios involving collapsed or heavily damaged buildings, the four reflection regions lose recognition (Brunner et al. 2010). The bright layover area and robust double-bounce line might partially or completely disappear (Gong et al. 2016; Wu et al. 2016). Building shapes deviate from regularity, as debris functions as corner reflectors, yielding localised strong reflections (Kuny et al. 2015). These observations suggests that the textural features within VHR SAR imagery have the potential to differentiate between damaged and undamaged structures.

**Fig. 1:**
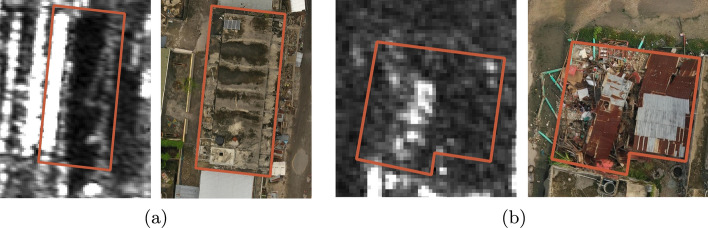
Building appearance in VHR SAR imagery and a drone orthophoto: **a** an intact building, and **b** a collapsed structure. The VHR SAR data is sourced from Capella imagery acquired over Les Cayes, Haiti (©2021 Capella Space, all rights reserved)

A visual representation of both intact and collapsed buildings in VHR SAR images is depicted in Fig. 1.

## Earthquake case studies

We tested the proposed method on two major earthquakes that occurred within the past two years: (1) the 2021 Nippes Earthquake in Haiti, and (2) the 2023 Kahramanmaraş earthquake sequence in Turkey. These earthquake events were chosen due to the substantial devastation, significant economical and humanitarian impact, and the availability of recent VHR SAR imagery data. Furthermore, the differences in damage pattern, spatial scales, and data availability allowed us to assess the flexibility and generalisability of the proposed method.

**Fig. 2:**
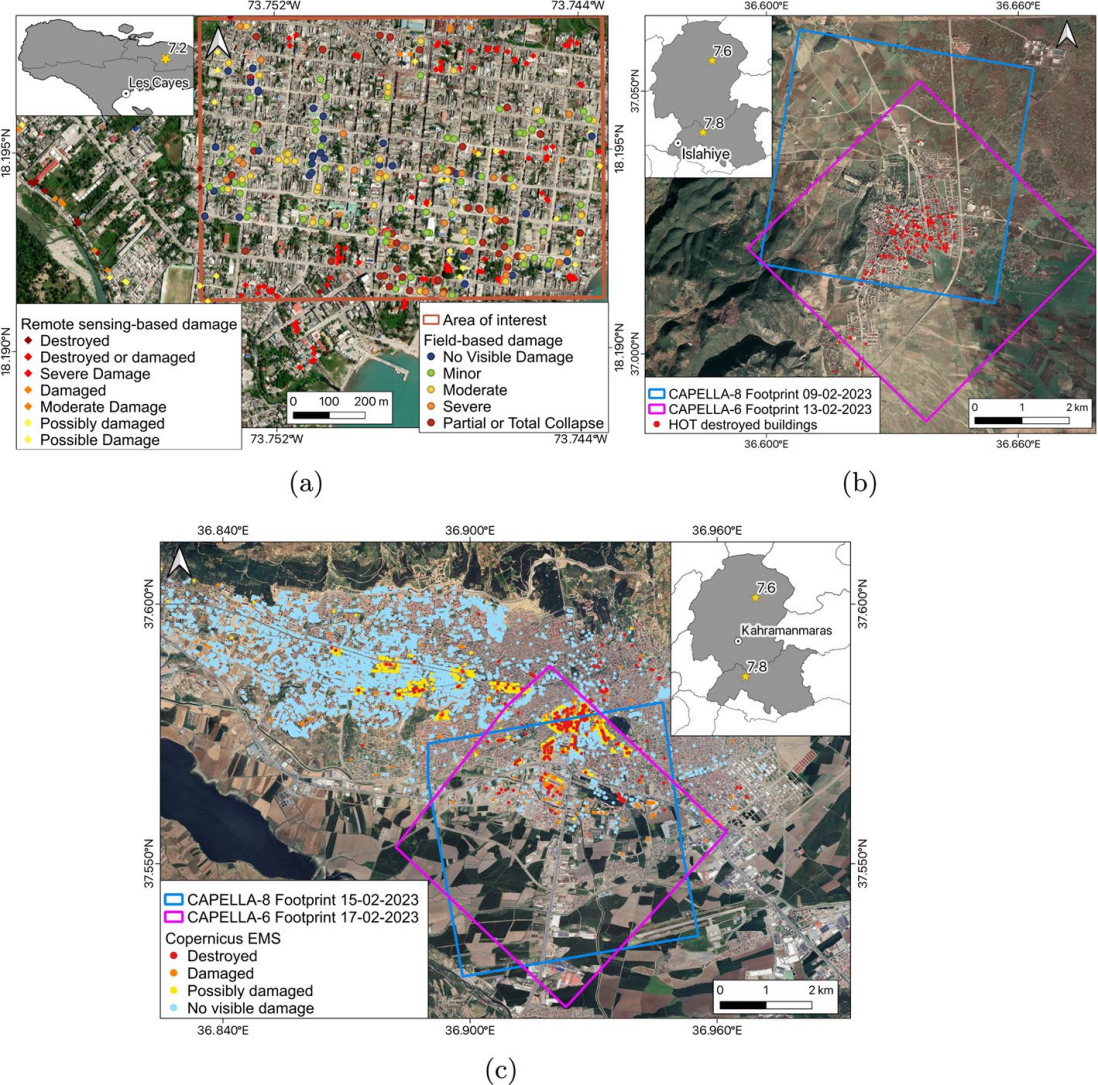
Study areas and distribution of building damage data in **a** Les Cayes, Haiti, **b** Islahiye Turkey, and **c** Kahramanmaraş, Turkey. Square polygons delineate the regions analysed for each case study, as defined in Table 1. In the location maps, star symbols indicate epicentres of the earthquake mainshocks and major aftershock

The location of the study areas for both earthquake events is shown in Fig. 2.

### The 2021 Nippes earthquake

On the 14th of August 2021, the Tiburon Peninsula in Haiti experienced a moment magnitude (M_w_) 7.2 earthquake. The epicentre was approximately 150 km west of the capital city, Port-au-Prince, with an hypocentre depth of around 19 km (Calais et al. 2022). The earthquake was followed by a substantial number of aftershocks, with over 2000 recorded (Douilly et al. 2022).

The South Department of Haiti suffered significant consequences. The confirmed death toll exceeded 2248, and more than 15,000 individuals were reported injured (UN OCHA 2021). The impact on buildings was severe, with over 137,500 structures destroyed or damaged. This earthquake stands as the deadliest event of 2021 and represents the most severe disaster to hit Haiti since the 2010 M_w_ 7.0 earthquake. One of the heavily affected urban areas was Les Cayes, the third-largest city in Haiti. Les Cayes witnessed extensive damage, including the collapse of residential homes, religious structures, and commercial buildings, and thus was selected as a case study for this research.

### The 2023 Kahramanmaraş earthquake sequence

On the 6th of February 2023 at 04:17 a.m. local time (01:17 a.m. UTC), southeastern Turkey and northern Syria experienced a M_w_ 7.8 earthquake. Approximately 9 h later, the main earthquake was followed by a M_w_ 7.6 aftershock (AFAD 2023). The epicentre of the main earthquake was located 26 km east of the city of Nurdagi, in Turkey’s Gaziantep province, with a focal depth of 8.6 km (Naddaf and Callaway 2023). The M_w_ 7.6 aftershock occurred around 4 km southeast of Ekinözü, in the Kahramanmaraş province. Following this doublet earthquake, more than 12,000 aftershocks continued to shake the devastated regions.

These earthquakes represent the strongest earthquake sequence in Turkey in more than 80 years and rank as the fifth-deadliest seismic event of the 21st century (Dal Zilio and Ampuero 2023). The resulting shaking impacted a wide region of southern Turkey and northern Syria, resulting in a loss of nearly 60,000 lives and the damage or destruction of hundreds of thousands of buildings. The primary and secondary effects included surface ruptures, ground cracks, liquefaction phenomena, and hydrological anomalies, leading to extensive damage to buildings and infrastructure networks. For our study, we analysed the areas of Islahiye and Kahramanmaraş, which were heavily impacted by the 2023 Kahramanmaraş earthquake sequence.

## Data

This section presents an overview of the data used in this study, including post-event VHR SAR imagery, building damage data with damage grade labels, and building footprint inventories. The selected study areas are characterised by a diverse range of environmental settings, structural typologies, type and distribution of damage, and varying image acquisition conditions. This diversity allows for a comprehensive representation of post-earthquake real-world scenarios, facilitating the development of a general model that could be applied to future disaster assessments.

### Post-event VHR SAR imagery

Five post-event VHR SAR images from the Capella satellite constellation were employed for the analysis of Les Cayes in Haiti, and Kahramanmaraş and Islahiye in Turkey. Table 1 provides details of the VHR SAR images used for each study area. The ‘letter code’ column refers to the naming convention adopted throughout the manuscript to unequivocally identify each case study.

The Capella satellites are equipped with X-band radar systems that enable the collection of VHR data in Spotlight mode (Stringham et al. 2019). Each SAR image utilised in this study covers an area of 25 km^2^, with range and azimuth resolutions ranging from 0.38 m to 0.88 m (Table 1). The availability of the SAR images varied, with some being publicly accessible through the Capella Space Open Data catalogue for disaster response and others being acquired through the Capella Space online platform.

All SAR images provided amplitude information only and were delivered as Geocoded Terrain Corrected (GEO) products (Capella Space 2022). Prior to delivery, the data underwent several preprocessing steps, including range-compression, detection, focusing, and multi-looking. Additionally, a high-resolution digital elevation model (DEM) was used to geocode the data and correct it for terrain height. The multi-looking process, with a factor of 1 $\times$ 9 (range $\times$ azimuth), was applied to enhance the radiometric resolution of the images, resulting in reduced noise and improved sensitivity to brightness changes.

**Table 1:** Main characteristics of the Capella VHR SAR data for the study areas in Haiti and Turkey

Study area	Letter code	Acquisition date	Satellite	Orbit	Look direction	Polaris.	Rg $\times$ az resolution (m $\times$ m)	Look angle (^∘^)
Les Cayes, Haiti	A	16/08/2021	Capella-5	Desc.	Right	HH	0.59 $\times$ 0.64	48.84
Islahiye, Turkey	B	09/02/2023	Capella-8	Desc.	Right	HH	0.38 $\times$ 0.64	39.20
Islahiye, Turkey	C	13/02/2023	Capella-6	Asc.	Left	HH	0.38 $\times$ 0.64	23.87
Kahramanmaraş, Turkey	D	15/02/2023	Capella-8	Asc.	Right	HH	0.44 $\times$ 0.88	33.22
Kahramanmaraş, Turkey	E	17/02/2023	Capella-6	Asc.	Left	HH	0.46 $\times$ 0.88	34.84

‘Polaris.’ stands for polarisation, ‘Asc.’ and ‘Desc.’ indicate ascending and descending orbits, respectively, and ‘Rg $\times$ az’ stands for range $\times$ azimuth resolution

### Building damage data

In operational scenarios, data from different sources with varying quality is usually available. This study benefits from the diversity of data sources, including field-based and remote-sensing-based building damage data, enhancing the generalisability of the proposed method.

#### Les Cayes, Haiti

For the Les Cayes study area in Haiti, the building damage data utilised in this research was provided by the Structural Extreme Event Reconnaissance (StEER) and GeoHazards International (GHI) teams. StEER/GHI mobilised local field data collectors, who were mainly non-engineers, to rapidly gather information on damage descriptors and capture geotagged photographs following the 2021 Haiti earthquake (Kijewski-Correa et al. 2022). The collected data was made accessible through the StEER Rapid Response (M7.2 Haiti EQ - Aug 2021) App in Fulcrum, facilitating a rapid seismic assessment. Experts, including the EEFIT team, remotely conducted the assessment by examining the photographic material and any other available information within the Fulcrum App (Whitworth et al. 2022). The assessment involved (1) classifying the structural typology into seven categories, including ‘reinforced concrete with infill masonry shear walls’ (RC), ‘confined masonry’ (CM), ‘unreinforced masonry bearing walls’ (URM), ‘reinforced masonry bearing walls’ (RM), ‘wood light frames’ (WL), ‘wood with stone infills’ (WS), and ‘unknown’ (UN), and (2) assigning a damage rating according to a five-level scale. This damage scale is an adapted version of the European Macroseismic Scale (EMS-98), as outlined in the StEER assessment manual (Miranda 2021), and includes the following classes: ‘no damage’ (D1), ‘minor damage’ (D2), ‘moderate damage’ (D3), ‘severe damage’ (D4), and ‘total or partial collapse’ (D5). In our study area in central Les Cayes, a total of 215 records out of the 11,669 assessed buildings were available (Fig. 2a).

In addition to the field-based building survey data, remote sensing-based damage records were also collected. These records consist of geocoded building centroids generated by the International Organization for Migration (IOM 2021). The data integrates satellite-based damage assessments carried out by Copernicus EMS and UNOSAT with photo interpretation of drone imagery. Each record is labelled with a damage level, including ‘Possible’ or ‘Possibly damaged’, ‘Moderate Damage’, ‘Damaged’, ‘Severe Damage’, ‘Destroyed or damaged’, and ‘Destroyed’ (Fig. 2a).

#### Islahiye, Turkey

For Islahiye, we used the building damage data produced by the Humanitarian OpenStreetMap Team (HOT), a digital humanitarian group involved in crowd-sourced remote damage assessments based on satellite imagery following major disasters (HOT 2023b). In response to the events of the 6th February 2023, HOT was activated to support the disaster response in central Turkey and northern Syria. The source data for the Turkey-Syria earthquake activation included aerial and satellite imagery from the Copernicus EMS and the Implementation and Research Centre for Satellite Communications and Remote Sensing at the Istanbul Technical University. By using these data sources, HOT manually identified collapsed buildings, and the resulting data consisted of maps and crowdsourced geospatial layers. The distribution of collapsed buildings in Islahiye is shown in Fig. 2b.

As an open crowd-sourcing project, HOT allows contributions from anyone to create and update maps and geospatial products. To ensure high-quality crowd-sourced data for emergencies such as the Turkey and Syria earthquake response, a four-step validation process was implemented (HOT 2023a).

#### Kahramanmaraş, Turkey

For Kahramanmaraş, we utilised the building damage data released by Copernicus EMS, which was activated in the aftermath of the Turkey–Syria earthquake sequence to aid in the damage assessment. For this activation, Copernicus EMS used sub-metre resolution optical satellite images between the 7th and 12th of February 2023, covering 20 areas of interest located near the earthquake epicentre (CEMS 2023). Due to cloud coverage in some areas, multiple satellite images were necessary to complete the assessment.

The acquired optical satellite images, primarily from Worldview-2 and Pleiades sensors, were used by Copernicus EMS to generate preliminary damage assessment maps for each study area. These mapping products were made available as graphical maps and vector data to be used within a geospatial information system (GIS) environment. All products underwent a quality assessment by the European Commission’s Joint Research Centre to ensure high-quality results. The damage assessment mostly focused on the city block level, but a more detailed analysis at the building-level was also available for some locations, including Kahramanmaraş.

In our study, we specifically used the vector data resulting from the building-level analysis. This data consists of geocoded points corresponding to damaged or suspected damaged buildings in the urban area of Kahramanmaraş (Fig. 2c), and also includes information on the processing method used for the assessment, as well as the level of damage of each structure. The damage scale used in the analysis adopts four levels: ‘no visible damage’, ‘possibly damaged’, ‘damaged’, and ‘destroyed’. This scale is a modified version of the EMS-98 and was introduced by Copernicus EMS to account for the challenges of remotely sensed imagery and time constraints of near-real-time damage assessment (Cotrufo et al. 2018; Inès et al. 2020). The ‘possibly damaged’ class accounts for uncertainty in the image analysis and interpretation, as well as damage not clearly visible from above. The ‘damaged’ class encompasses damage levels from negligible to substantial, while the ‘destroyed’ class represents very heavy damage and collapses.

It is worth noting that the analysis of the satellite imagery in this dataset was conducted using a photo-interpretation detection method, involving manual analysis without the support of automatic processes. This makes the dataset suitable as an input for ML classification algorithms.

### Building footprint data

For the Les Cayes study area in Haiti, building footprints were digitised manually in a GIS environment for a specific region in central Les Cayes (Fig. 2a). To perform this task, a post-event orthophoto with a resolution of 2 cm, acquired by an aerial drone on the 18th of August 2021, was utilised. This orthophoto was released by HaitiData (HaitiData 2021), a web-based platform developed after the 2010 Haiti earthquake to disseminate GIS and other cartographic data in support of disaster management. To account for the shape of totally or partially collapsed buildings, an additional drone orthophoto with an 8 cm resolution, dating back to October 2016, was also employed. The combined use of these orthophotos facilitated the mapping of a total of 4,116 building footprints (Giardina et al. 2023).

For both Islahiye and Kahramanmaraş in Turkey, we used the Microsoft building footprints released as part of the Global ML Building Footprints program (Microsoft 2023). This data is freely available and consists of geocoded building footprints derived from Bing imagery between 2014 and 2023 through artificial intelligence and computer vision tools.

## Method

The proposed method draws inspiration from object-based analysis in classifying building damage from post-event VHR SAR imagery. This method leverages the spatial relationships and characteristics of building segments in the amplitude and textural features derived from post-event radar images to carry out a rapid damage assessment after seismic events. Building footprints are used as image objects, as they align with the inherent structural units of the built environment.

The method involves a step-by-step process that begins with the extraction of textural features from post-earthquake VHR SAR imagery (Sect. 5.1.1). Specifically, grey level co-occurrence matrix (GLCM) textural features based on second-order statistics were calculated. These features aim to capture unique characteristics of individual damaged and undamaged buildings, forming the basis for modelling the relationship between the post-event SAR imagery and the building damage data through supervised ML. The next step involves the segmentation of the raster layer to be classified (Sect. 5.1.2), utilising building footprints as image objects. This segmentation allows for a systematic examination of the textural properties of the segments, facilitating the identification of damage patterns at the building level. To classify the building damage, a random forest (RF) classifier is trained (Sect. 5.2.1) using the building damage data described in Sect. 4.2 and associated statistics. The classifier assigns a label indicating the level of building damage to each segment, based on a binary classification system, i.e., ‘standing’ and ‘collapsed’ (Sect. 5.1.3). The RF classifier is chosen for its suitability in handling complex classification tasks and providing accurate results, especially with high-dimensional data (Geiß et al. 2015).

**Fig. 3:**
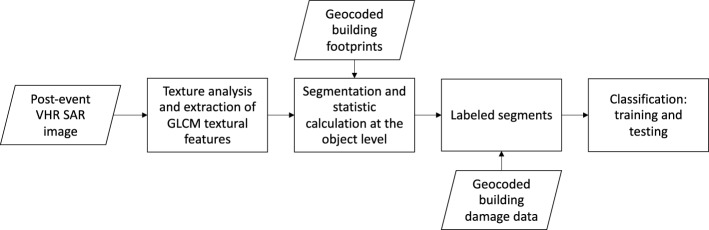
Flowchart of the step-by-step methodology

The next sections provide a detailed description of the procedure for preparing the training and testing datasets (Sect. 5.1), as well as the implementation of the classification process (Sect. 5.2). A workflow picture illustrating the method is shown in Fig. 3.

### Data preparation

In the data preparation stage, we conducted various steps to ensure the suitability and consistency of the input data for the subsequent classification tasks. This involved textural analysis and feature extraction, segmentation and attribute calculation, and pre-processing of the labelled data.

#### Textural analysis and feature extraction

The first step was to perform textural analysis on the post-event Capella SAR images for each study area. Specifically, we utilised the SNAP Sentinel-1 toolbox (SNAP 2022) to extract GLCM textural features (Haralick et al. 1973) from the amplitude data of each post-event Capella SAR image. A window size of 15 $\times$ 15 pixels was chosen for the textural analysis. This window size was selected based on the high resolution of the data, which is approximately 50 $\times$ 50 cm in range and azimuth, and aligns with the average size of buildings in the study area. The selected window size is also consistent with previous studies conducted in the field (Kuny and Schulz 2014; Gong et al. 2016). Since the directionality of textures was not relevant for our study, results from different directions, i.e., 0^∘^, 45^∘^, 90^∘^, and 135^∘^, were averaged to obtain the final textural features. Ten textural features, including contrast, dissimilarity, homogeneity, angular second moment (ASM), energy, maximum probability (MAX), entropy, mean, variance, and correlation, were estimated for each Capella image. These textural features, along with the amplitude data, were combined to create a raster stack for each Capella SAR image, providing a multi-dimensional view of the post-event Capella SAR imagery for subsequent analysis and classification tasks.

#### Segmentation and attribute calculation

To classify the damage at the individual building level, we implemented an object-scale classification approach using the building footprint data described in Sect. 4.3. For each case study, we employed the building footprint layers as a basis for segmenting the raster stacks generated in the previous step. This segmentation approach can be replicated in scenarios where inventories of building footprints are available from local cadastral databases, or collaborative open projects such as OSM (2023) or Microsoft (2023). Alternatively, building objects can be identified from pre-seismic optical images using segmentation algorithms (Chini et al. 2014). Within each building footprint, which represents an image object, we extracted the pixels corresponding to the textural features calculated in the previous step (Sect. 5.1.1). For each textural feature, we computed statistics at the object scale, including mean, standard deviation, minimum, and maximum, to summarise the texture of each building object. These statistics serve the purpose of generating feature vectors that provide a compact and informative description of the texture in each object. Instead of using pixel values, which might be numerous and may not capture the essential characteristics, these statistics condense the information into a set of four values for each texture. As a result, each building object is represented by a 4 $\times$ 11-dimensional feature vector (4 values per each texture and 4 values for the amplitude data), capturing the distribution and characteristics of the textural features within each building object and aiding in the subsequent damage classification process.

#### Pre-processing of labelled data

The building damage data used in this study was collected during two different earthquake events from various data sources (Sect. 4.2). Furthermore, this data was labelled according to different damage scales and criteria, resulting in an inconsistent number of classes that were not directly comparable. To ensure consistency in the labelled data for training purpose, some pre-processing was undertaken.

**Fig. 4:**
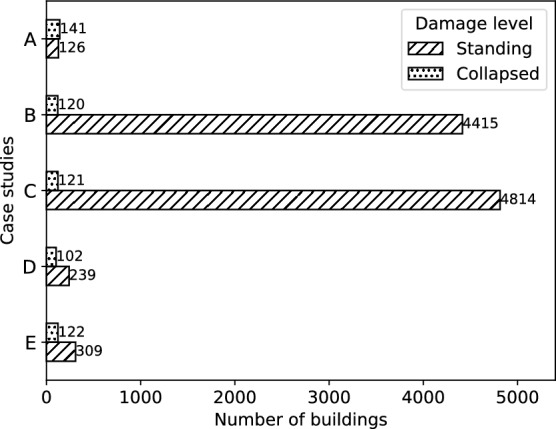
Distribution of building damage data per study area based on a binary damage classification system

Considering the satellite perspective, which primarily allows the distinction between standing and collapsed structures (Huynh et al. 2014; Cotrufo et al. 2018), a binary classification system was adopted. Specifically, for the Les Cayes study area in Haiti, where the field data followed a five-level damage scale, the ‘severe damage’ class was excluded from the study. Despite technically falling under the ‘standing’ category, buildings with severe damage might exhibit unique backscattering patterns that could potentially lead to confusion in classification categories. Moreover, our primary focus was on distinguishing between standing and collapsed buildings, aligning with the urgent needs of disaster response scenarios. As a result, the decision not to merge ‘severe damage’ with ‘standing’ was made to maintain a clear distinction between intact structures and those significantly impacted by the earthquake. The structures labelled as ‘no damage’, ‘minor damage’, and ‘moderate damage’ were combined into a single class and relabelled as ‘standing’. The buildings labelled as ‘total or partial collapse’ were combined with the ‘destroyed’ and ‘destroyed or damaged’ buildings obtained from the remote sensing-derived data for the same study area, and relabelled as ‘collapsed’. Similarly, for Kahramanmaraş in Turkey, we utilised only the damage records labelled as ‘no visible damage’ or ‘destroyed’. For Islahiye in Turkey, where only ‘collapsed’ buildings were available, undamaged structures were obtained by extracting the difference between the geocoded building footprints and the collapsed ones. Subsequently, the classes were renamed as ‘standing’ and ‘collapsed’ for all case studies. Figure 4 shows the number of labelled damaged buildings for each study area after applying these class adjustments.

Then, the labelled records located within the extents of the corresponding Capella images were extracted for each study area from A to E. Finally, the statistics computed in the previous step (Sect. 5.1.2), were assigned to the corresponding labelled records within each building object.

### Training process

To build a generalised RF model for classifying standing and collapsed buildings using post-event VHR SAR images and derived textural features in different post-earthquake scenarios, we designed a three-level training approach. The data was randomly split into 80% for training and 20% for testing purposes for each study area. The training process involved:

1. Standard supervised learning: initially we implemented a standard supervised learning workflow by training a model for each case study using the corresponding training dataset. The trained model was then used to classify building damage within the same study area.
2. Combined learning: subsequently, we proposed a combined learning approach that used combined inventories from multiple case studies to build a common model. By incorporating data from different scenarios, the model could learn from a broader range of building damage patterns and improve its classification performance.
3. Generalisation testing: finally, we assessed the generalisability of the RF model by testing it on a new study area that was not encountered during the training process. This step allowed us to assess the model’s ability to classify building damage in unfamiliar contexts and evaluate its robustness and adaptability for operational scenarios where training data may not be available.

The next sections provide details on the chosen classifier, the strategies employed to address data imbalance, and the evaluation metrics applied in this study to assess the classification performance.

#### Random forest

The RF algorithm is a ML technique used for classification tasks (Breiman 2001). Unlike parametric classifiers, RF learns the relationship between the training data and the response dataset, without starting with a predefined data model. It employs an aggregated model that combines the outputs of multiple decision trees to calculate the response variable. Each decision tree in the RF is constructed using a subset of the training samples, selected with replacement (bagging approach), and a random selection of variables at each node. The best split based on these variables is chosen to create two sub-nodes. By combining these trees, the RF ensemble is formed. The classification of each image object is determined by the votes from all trees, with the final class being the one receiving the maximum number of votes. RF offers several advantages, including robustness to outliers and noise, estimates of error and variable importance, and the ability to handle a large number of variables. Furthermore, it overcomes the overfitting problem of decision trees. RF has proven to be a powerful classifier for detecting damaged buildings in satellite imagery (Cooner et al. 2016; Ji et al. 2019).

In this study, RF was selected as the classifier to identify collapsed and standing buildings. The decision to use this nonparametric classifier was made considering the presence of possible feature redundancy, which can pose challenges for accurately estimating statistics in parametric approaches (Geiß et al. 2015). The training process was implemented using the scikit-learn Python library (Pedregosa et al. 2011). The RF hyperparameters include the number of trees to grow and the number of features used at each node. These hyperparameters were evaluated by measuring the RF out-of-bag (OOB) error and finding the computationally optimal number of trees. To determine suitable parameter values, multiple trials were conducted, and it was observed that a convergence point was reached at a specific number of trees. In this study, a value of 400 trees was chosen for reliable OOB error estimation. The number of features used at each node was determined by evaluating different values, and we found that the best performance was achieved when the full set of predictors was used.

#### Imbalanced classes

To address data imbalance arising from the unequal distribution of labelled buildings in the training dataset, we employed techniques specifically designed to handle imbalanced data. These techniques aim to ensure that the model is not biased towards the majority class and can accurately classify both collapsed and standing buildings.

Common methods for addressing class imbalance include random downsampling, random oversampling, and data augmentation techniques such as the synthetic minority oversampling technique (SMOTE). Random downsampling involves the random removal of instances from the majority class to align with the minority class. This technique prevents the overwhelming influence of the majority class during the learning process. Random oversampling is the process of randomly generating new instances from the minority class to balance its representation within the majority class. One approach to oversampling involves duplicating samples from the minority class within the training dataset through resampling with replacement. An improvement to oversampling with replacement involves generating new synthetic samples from the minority class using the SMOTE technique (Chawla et al. 2002). With SMOTE, a random sample from the minority class is first chosen. Then, *k*-nearest neighbours are identified (typically *k*=5) for that sample. From these neighbours, one is randomly selected, and a synthetic sample is generated at a randomly selected point between the two samples. This procedure can be repeated to create a desired number of synthetic samples for the minority class. However, a drawback of this approach is that synthetic samples are generated without considering the majority class, potentially leading to ambiguous samples in cases of substantial overlap between classes.

Figure  4 shows that the distribution of standing and collapsed buildings per study area is imbalanced, with most case studies containing a higher number of records for undamaged structures. This class imbalance is particularly evident in study areas B and C, where only the 2.67% and 2.45% of the total buildings, respectively, are labelled as collapsed. To mitigate the impact of class imbalance, we conducted multiple tests involving random downsampling, random oversampling, and SMOTE. Through such evaluation, we determined that the optimal performance was achieved when employing random downsampling. Consequently, the downsampling technique was adopted to the datasets from study areas A to E, allowing for a balanced representation of the two classes during model training.

#### Performance evaluation

The performance of the RF model’s predictions was quantified based on the accuracy of building labelling in each case study. The evaluation metrics used to assess the classification accuracy of the model included overall accuracy (OA), precision, recall, and cohen’s kappa coefficient (Kappa). For each metric, the corresponding mathematical equation is reported in Table 2. OA represents the percentage of buildings correctly classified as standing or collapsed. Precision indicates the proportion of correctly classified collapsed buildings, i.e., true positives (TP), to the sum of correctly classified collapsed buildings and standing buildings misclassified as collapsed, i.e., false positives (FP). Precision quantifies the classifier’s ability to avoid labelling undamaged buildings as collapsed. Recall measures the ratio of correctly classified collapsed buildings to the sum of correctly classified collapsed buildings and collapsed buildings misclassified as standing, i.e. false negatives (FN). Recall assesses the classifier’s ability to find all collapsed buildings. Undamaged buildings correctly classified as standing are referred to as true negatives (TN). Kappa is a statistical measure used to evaluate the level of agreement between raters for categorical variables. It is considered a robust measure as it accounts for the possibility of agreement occurring by chance. Kappa is particularly well suited for handling imbalance datasets, as it considers both overall accuracies and corresponding misclassification costs. Kappa values between 0.01 and 0.20 indicate slight agreement, 0.21–0.40 represent a fair agreement, 0.41–0.60 suggest moderate agreement, 0.61–0.80 indicate substantial agreement, and 0.81–1.00 signify almost perfect agreement (Landis and Koch 1977).

**Table 2:** Evaluation metrics used to assess the performance of the trained RF models

Metric score	Formula
OA	$\mathrm {\frac{TP + TN}{TP+TN+FP+FN}}$
Precision	$\mathrm {\frac{TP}{TP+FP}}$
Recall	$\mathrm {\frac{TP}{TP+FN}}$
Kappa	$\mathrm {\frac{2 \times (TP \times TN - FN x FP)}{(TP + FP) \times (FP + TN) + (TP + FN) x (FN + TN)}}$

## Results

We assessed the performance of the proposed method on three urban areas affected by two different earthquake events, utilising various sources of building damage data and VHR SAR imagery acquired on different dates and under varying imaging conditions. This led to the availability of five distinct case studies (Table 1). We conducted a three-level training approach, involving the training of twenty RF models for each level. Before each new training analysis, random splitting of training and testing sets, as well as random down-sampling of the datasets were performed. This ensured that the model remained unbiased toward the majority class, while simultaneously introducing variability by selecting a different subset of the majority class randomly for each iteration of training and testing, thereby minimising information loss.

The next sections summarise the results obtained for both the standard and combined learning approaches, as well as the generalisation test.

### Performance evaluation of a case study-based learning model

We trained twenty RF models on each labelled inventory from study areas A to E. For each classification analysis, data were randomly split into 80% training and 20% testing. When repeating the classification on a given area, we re-split the data to capture more general conditions as accurately as possible.

**Fig. 5:**
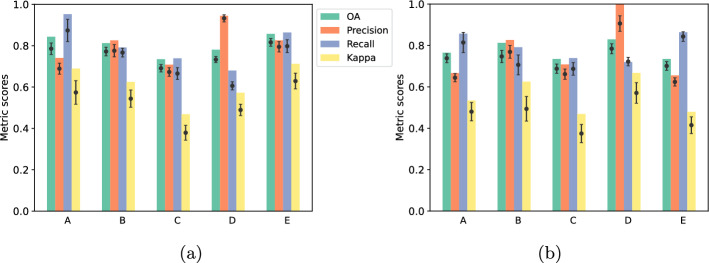
Comparison of OA, precision, recall and kappa metric scores for the five case studies using **a** a separate model for each study area and **b** a combined model. Downsampling was applied to address imbalanced classes. The dot within each bin indicates the average performance across twenty tests. Vertical error bars represent the standard deviations for each metric

Figure 5a shows the classification results obtained for the case study-based learning approach after applying downsampling, where the majority class was randomly downsampled to match the minority class for each case study (Sect. 5.2.2). The figure presents the metric scores, i.e., OA, precision, recall and kappa, for the best performing model in each case study, as well as the average performance and the standard deviation of the models trained across twenty tests, indicated by a black dot and a vertical bar, respectively.

Overall, the RF classifier demonstrated its ability to learn the features necessary to distinguish between standing and collapsed buildings within their respective testing regions. For every case study, the best performing model produced an OA higher than 70%, with the lowest performance observed for case study C, which produced the best score of 73%. Case studies A, B, and E showed the best performances, with an OA of 84%, 81% and 86%, respectively. In terms of Kappa, we found moderate to substantial agreement, with the lowest score of 0.47 for case study C and the highest score of 0.71 for case study E.

Precision values indicated the model ability to correctly identify a building as collapsed out of all the structures classified as collapsed, with only minimal chance of misclassifying a standing building as collapsed. Specifically, Precision values ranged from 71% in case study C to 94% in case study D. Similarly, recall values ranged from 68% (case study D) to 95% (case study A), indicating the model good ability to correctly identify damaged buildings. The results also demonstrate that the models trained on case studies B, C and E performed similarly in terms of precision and recall. On the other hand, the model trained on case study A exhibited excellent recall, but a relative lower precision, while the model trained on case study D achieved a very high precision but a lower recall.

### Performance evaluation of a combined learning model

In addition to the case study-based learning, we also explored a combined learning approach by combining the training data from the five different case studies analysed individually in the previous section. The labelled data was randomly divided into 80% training and 20% testing sets, and similarly to before, we applied downsampling to address class imbalance. Subsequently, we merged the training data from case studies A to E and employed this combined dataset to train twenty RF models.

Figure 5b shows the classification results obtained after applying downsampling. It can be observed that RF models trained on the combined inventories (Fig. 5b) exhibited similar performance to those trained using the case study-based learning approach (Fig. 5a). The best performing combined model achieved OA ranging from 73 to 83%, with case studies C and D demonstrating the lowest and highest accuracies, respectively. Moreover, kappa values ranged from 0.47 to 0.67, indicating moderate to substantial agreement. The lowest Precision was observed for case study E, scoring 66%, while case study D achieved perfect Precision with a score of 100%. Recall values ranged from 72% for case study D to 86% for case study A, demonstrating the model strong capability to correctly identify collapsed structures.

**Fig. 6:**
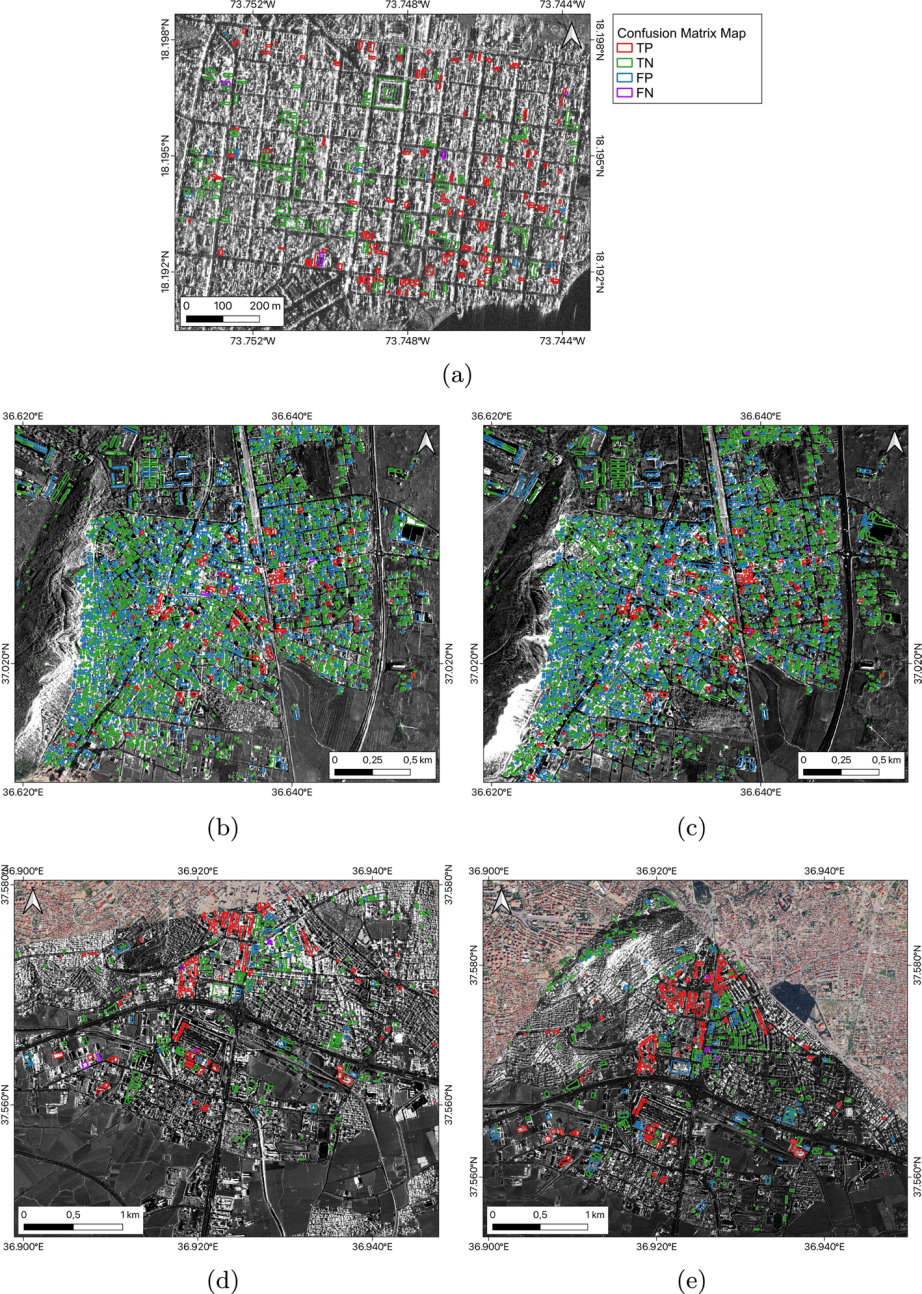
Confusion matrix maps: performance of the best combined models on full datasets for case studies **a** A, **b** B, **c** C, **d** D, and **e** E. Background data is the Capella VHR SAR imagery defined in Table 1 (©2023 Capella Space All Rights Reserved)

Figure 6 shows the confusion matrix maps generated by the best performing combined models when applied to the full datasets from case studies A to E. These confusion matrix maps display the predictions of TP, FP, FN, and TN for each case study.

### Performance generalisation

To test the generalisability of the combined learning model, the RF models were evaluated on a new environmental setting without using a local training inventory. To mitigate the impact of potentially inadequate inventory on the performance of the combined learning approach, we used the Jackknife’s, or leave-one-out, approach. This involved systematically excluding the inventory of one case study from the training data and utilising the complete inventories from the remaining case studies to predict damage for the excluded case study. The entire extent of the excluded case study was then designated as the testing area for evaluating the generalisation ability of the trained models. This procedure was repeated for each case study.

**Fig. 7:**
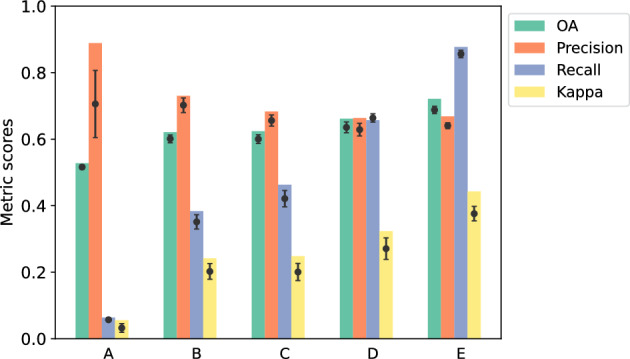
Comparison of OA, precision, recall and kappa metric scores for the five case studies using generalised trained RF models in a new environmental setting. The models were evaluated using inventories from the other four case studies as training data, with downsampling applied to address imbalanced classes. The dot within each bin represents the average performance across twenty tests. Vertical error bars denote the standard deviations for each metric

Figure 7 presents the performance comparison of the trained RF models in classifying damage within a specific study area, using inventories from the other four case studies as training data. The figure displays the best and average scores for the performance metrics, including OA, Precision, Recall, and Kappa, across twenty tests. This generalisation approach produced OA ranging from 53 to 72% for the best performing models, with average OA of 63%. Case study E exhibited a kappa 0.44, indicating moderate agreement, while case study A demonstrated the lowest performance with a Kappa of 0.1. The average Kappa across the five case studies was 0.26.

Precision values ranged from 66% (case study D) to 89% (case study A), with an average Precision of 73%, indicating the model ability to accurately identify damaged structures. Precision focuses on the exactness of positive predictions, representing the proportion of correctly predicted positive instances out of all predicted positives. However, the recall values were significantly lower, with case study A showing the lowest score. Recall measures the completeness of positive predictions, indicating the proportion of actual positive instances correctly identified by the model. In the case of case study A, the model missed several instances of actual damage. Specifically, Recall values ranged from 6% (case study A) to 88% (case study E), with an average recall of 49%. It is worth noting that for three case studies, the mapping of the RF models on unseen regions exhibited a bias towards high precision but relatively low recall. This indicates that the models were able to accurately identify damaged structures, i.e., high precision, but missed several instances of actual damage, i.e. low recall, in those case studies.

## Discussion

The primary goal of this study was to evaluate the feasibility of distinguishing between standing and collapsed buildings using available VHR SAR data and ML techniques. In comparison to traditional SAR approaches in post-earthquake damage assessment, which rely on the availability of pre-disaster SAR images, the proposed method is able to assess seismic damage based solely on post-event SAR data. This feature makes our approach particularly suitable for scenarios where pre-event SAR imagery is limited. Additionally, our method achieves damage classification at the individual building level, surpassing the limitations of existing post-event SAR methods that predominantly focus on the city block level.

The proposed methodology is designed to be flexible, accommodating also scenarios where only limited imagery is accessible. This is why we initially employed a standard supervised learning workflow (Sect. 6.1), tailoring models to specific case studies using the corresponding datasets for training. Conversely, the combined learning approach (Sect. 6.2) leverages inventories from multiple case studies to build a common model, providing a broader understanding of building damage patterns across different scenarios. Both standard and combined learning approaches demonstrated comparable performance metrics, highlighting their effectiveness in capturing relevant information for damage classification. The decision to use either standard or combined learning may ultimately depend on the specific goals of the analysis and the availability of data. While standard learning is tailored to individual case studies, combined learning offers a more generalised approach suitable for scenarios with limited labelled data or when aiming for broader applicability across multiple regions.

The generalisation test (Sect. 6.3) revealed lower performance metrics compared to the standard and combined learning approaches, particularly in terms of kappa. Factors affecting the performance of kappa can include the variation in damage rates across different regions and the simplification to aggregate labels into binary categories, considering diverse damage categories across case studies from different data sources (field-based and remote-sensed). The binary classification simplification, driven by the diversity of data sources available and a focus on rapid disaster response, may reduce kappa sensitivity to damage severity variations across different regions and events, particularly because the exclusion of severely damaged buildings was possible for the Haiti case study only. In other case studies, where labelled data is derived from remote sensing sources, there is a possibility that some severely damaged buildings have been included in either of the two labels. Additionally, given the differences inherent in the various data sources used, their reliability is naturally expected to differ as well, potentially affecting generalisation capabilities across cases studies. The field data, obtained through on-site inspections, naturally offer a more comprehensive and detailed evaluation of damage due to the close proximity offered by in-person evaluations. Moreover, since the assessment was conducted by trained engineers, high reliability is expected for the damage scoring. For the HOT project, crowdsourced data utilising remote sensing imagery was conducted by individuals with different backgrounds and experiences. Furthermore, the assessment reliability is highly dependent on the quality and resolution of remote sensing imagery utilised. However, these differences reflect the diverse data availability and quality typical in operational scenarios. Finally, the variation in damage rates across different regions is another factor that may impact the generalisation of the kappa agreement score. The inherent differences in building construction, local seismic vulnerabilities, and other regional factors can contribute to varying rates of damage in response to earthquakes, and different backscattering mechanisms in the SAR imagery data.

When considering the method application in disaster response scenarios, the choice between precision and recall becomes a critical consideration. Precision minimises false positives, ensuring that identified collapsed buildings are actually damaged. On the other hand, recall focuses on identifying as many collapsed buildings as possible, even at the cost of potential false positives. The relative importance of precision and recall varies based on response goals and available resources.

The inclusion of data sources from different regions and earthquake events allowed us to assess the RF model sensitivity to varying geographical areas. Additionally, by utilising VHR SAR imagery with varying imaging characteristics and acquisition time points after the earthquake, the separation of case studies B and C, as well as D and E, despite covering nearly identical areas over Islahiye and Kahramanmaraş, serves to highlight situations where specific imaging conditions contribute to a more accurate assessment. Since the same structure might exhibit different backscattering characteristics under diverse imaging conditions, this separation also aids in training the classifier to recognise that similar types of damage can manifest differently in terms of backscattering. Consequently, maintaining the separation of these case studies provides an opportunity to emphasise individual strengths and challenges in specific contexts. Since the overlap between the datasets over regions B and C, as well as D and E is not perfect, and we implemented random downsampling and random splitting between training and testing for each analysis, it is unlikely that exact same datasets are used for training and testing over B and C or D and E. Consequently, considering this subtle variation in the building damage data and the expected different backscattering mechanisms, we expect to minimise the risk of bias in the final classification.

While our current method focuses on a binary classification for immediate post-earthquake damage assessment, future work could explore modifications to accommodate additional damage levels, adopting, for example, a traffic light-based damage classification system. However, this extension would require careful consideration of trade-offs between model complexity, computational efficiency, the inherent limitation of satellite perspectives, and the availability of labelled data for training purposes. Future work can also focus on further improving the method generalisation ability and exploring additional features to enhance the classification accuracy. This may involve refining existing model parameters and potentially exploring advanced machine learning and deep learning techniques to further enhance classification capabilities.

## Conclusion

In this paper, we introduced a novel method for classifying earthquake-induced building damage based solely on post-event VHR SAR imagery. The proposed method combines amplitude data and textural features extracted from post-event radar images with ML techniques, enabling rapid, regional-scale damage assessment following seismic events. The proposed method employed a three-level training approach. Initially, we trained supervised learning models on individual case studies using corresponding datasets for training and validation. Subsequently, we trained a combined learning model that incorporated inventories from multiple case studies to improve generalisability. Finally, we assessed the method flexibility by testing the ML model on a new study area, evaluating its adaptability to unfamiliar contexts where training data may be limited. As our method relies on the satellite perspective, which primarily allows differentiation between standing and collapsed structures, we implemented a binary classification system. The output includes damage maps that could assist stakeholders and decision makers in post-earthquake scenarios.

We evaluated the proposed method on three different urban areas that were affected by two recent earthquake events: the 2021 Nippes earthquake and the 2023 Kahramanmaraş earthquake sequence. We utilised five post-event VHR Capella SAR images obtained under varying imaging conditions. The analysis encompassed five distinct case studies, each exhibiting unique imagery characteristics, damage patterns, and spatial scales. Additionally, we had access to diverse sources of building damage records for both training and validation purposes, enabling us to evaluate the flexibility and generalisability of the proposed method. This diverse range of scenarios provided a comprehensive representation of real-world post-earthquake situations, facilitating the development of a robust model for future disaster assessments.

The following conclusions can be drawn:

- The proposed method allows for regional-scale damage assessment at the building-level through post-event VHR SAR imagery solely, enabling the rapid distinction between standing and collapsed buildings in the aftermath of an earthquake event.
- The proposed method demonstrates promising results in classifying standing and collapsed buildings in different post-earthquake scenarios, demonstrating that it is not restricted to a specific input dataset.
- The RF models trained on individual case studies achieved satisfactory performance, with OAs ranging from 73 to 86%. The models showed moderate to substantial agreement, as indicated by Kappa values ranging from 0.47 to 0.71. Precision values reflected the model ability to correctly identify collapsed buildings, while Recall values highlighted their capability to accurately identify damaged structures.
- The RF models trained on the combined datasets demonstrated performance similar to those trained using the case study-based learning approach. The best-performing combined model achieved OAs ranging from 73 to 83%, with moderate to substantial agreement, as indicated by the Kappa values ranging from 0.47 to 0.67. Precision and Recall values showed consistent patterns with the case study-based models.
- The RF models tested on a new environmental setting demonstrated an average OA of 63%, indicating reasonable generalisation ability. However, Precision values remained high, while recall values were comparatively lower. This suggests that the models accurately identified damaged structures but missed several instances of actual damage in unseen regions.

The proposed method offers a promising solution for classifying earthquake-induced building damage using post-event VHR SAR imagery. By integrating amplitude data, textural features, and ML techniques, we achieved accurate damage assessment across diverse urban areas affected by different earthquake events. This approach opens up the potential for near-real-time damage assessment, providing a valuable complement to in-field reconnaissance missions, while overcoming the limitations of weather and day-light dependent remote sensing techniques, such as those relying on optical imagery.

## Supplementary Information

Below is the link to the electronic supplementary material.

Supplementary file 1 (pdf 394 KB)
