# Supplementary material for: Integrating post-event very high resolution SAR imagery and machine learning for building-level earthquake damage assessment
Source: Bull Earthq Eng. 2024 Mar 9;23(12):5021–47. doi: 10.1007/s10518-024-01877-1 (PMC12457488; doi:10.1007/s10518-024-01877-1)
Supplement: Supplementary file 1 — (pdf 394 KB) [file 10518_2024_1877_MOESM1_ESM.pdf]

Supplementary Information:

Table S.1: Summary of features used for ML analysis.

Feature Number	Feature Name
Feature 1	SAR amplitude (mean value)
Feature 2	SAR amplitude (standard deviation value)
Feature 3	SAR amplitude (maximum value)
Feature 4	SAR amplitude (minimum value)
Feature 5	Contrast texture (mean value)
Feature 6	Contrast texture (standard deviation value)
Feature 7	Contrast texture (maximum value)
Feature 8	Contrast texture (minimum value)
Feature 9	Dissimilarity texture (mean value)
Feature 10	Dissimilarity texture (standard deviation value)
Feature 11	Dissimilarity texture (maximum value)
Feature 12	Dissimilarity texture (minimum value)
Feature 13	Homogeneity texture (mean value)
Feature 14	Homogeneity texture (standard deviation value)
Feature 15	Homogeneity texture (maximum value)
Feature 16	Homogeneity texture (minimum value)
Feature 17	Angular Second Moment (ASM) texture (mean value)
Feature 18	Angular Second Moment (ASM) texture (standard deviation value)
Feature 19	Angular Second Moment (ASM) texture (maximum value)
Feature 20	Angular Second Moment (ASM) texture (minimum value)
Feature 21	Energy texture (mean value)
Feature 22	Energy texture (standard deviation value)
Feature 23	Energy texture (maximum value)
Feature 24	Energy texture (minimum value)
Feature 25	Maximum probability (MAX) texture (mean value)
Feature 26	Maximum probability (MAX) texture (standard deviation value)
Feature 27	Maximum probability (MAX) texture (maximum value)
Feature 28	Maximum probability (MAX) texture (minimum value)
Feature 29	Entropy texture (mean value)
Feature 30	Entropy texture (standard deviation value)
Feature 31	Entropy texture (maximum value)
Feature 32	Entropy texture (minimum value)
Feature 33	Mean texture (mean value)
Feature 34	Mean texture (standard deviation value)
Feature 35	Mean texture (maximum value)
Feature 36	Mean texture (minimum value)
Feature 37	Variance texture (mean value)
Feature 38	Variance texture (standard deviation value)
Feature 39	Variance texture (maximum value)
Feature 40	Variance texture (minimum value)
Feature 41	Correlation texture (mean value)
Feature 42	Correlation texture (standard deviation value)
Feature 43	Correlation texture (maximum value)
Feature 44	Correlation texture (minimum value)

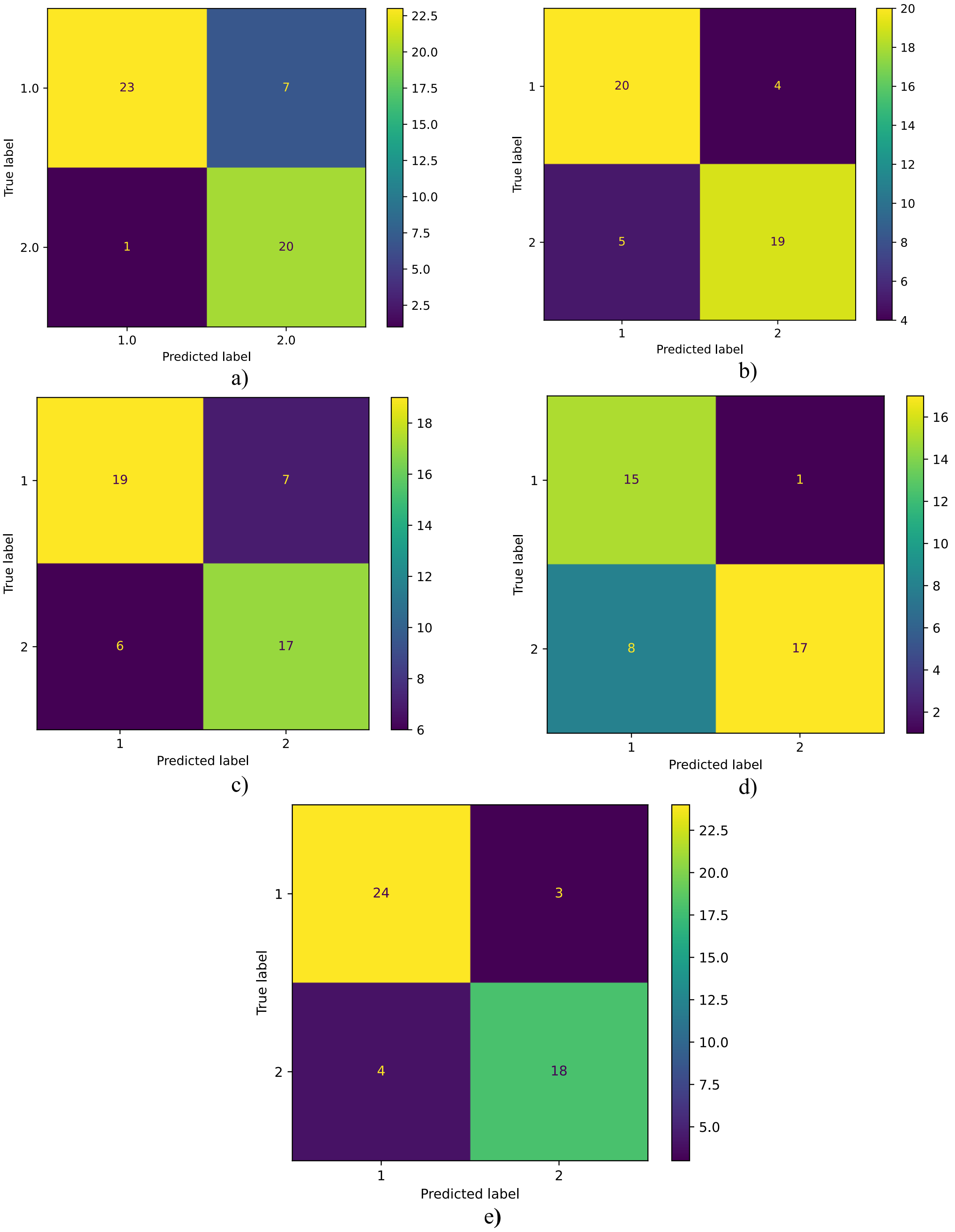

Figure S.1: Confusion matrices for the best performing models across twenty tests derived from the standard learning approach for case studies (a) A, (b) B, (c) C, (d) D, and (e) E. Labels '1' and '2' correspond to the 'Standing' and 'Collapsed' categories, respectively.

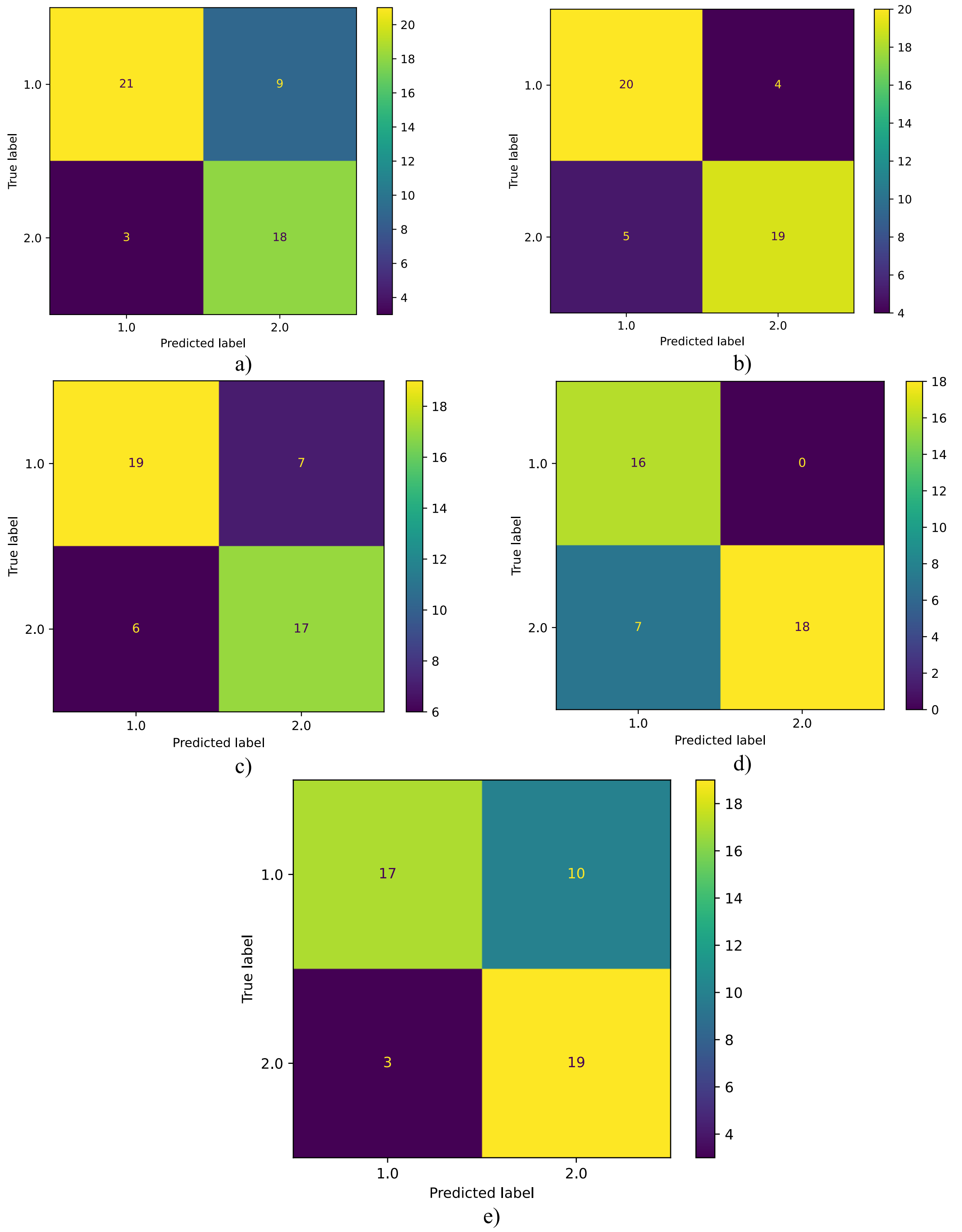

Figure S.2: Confusion matrices for the best performing models across twenty tests derived from the combined learning approach for case studies (a) A, (b) B, (c) C, (d) D, and (e) E. Labels '1' and '2' correspond to the 'Standing' and 'Collapsed' categories, respectively.

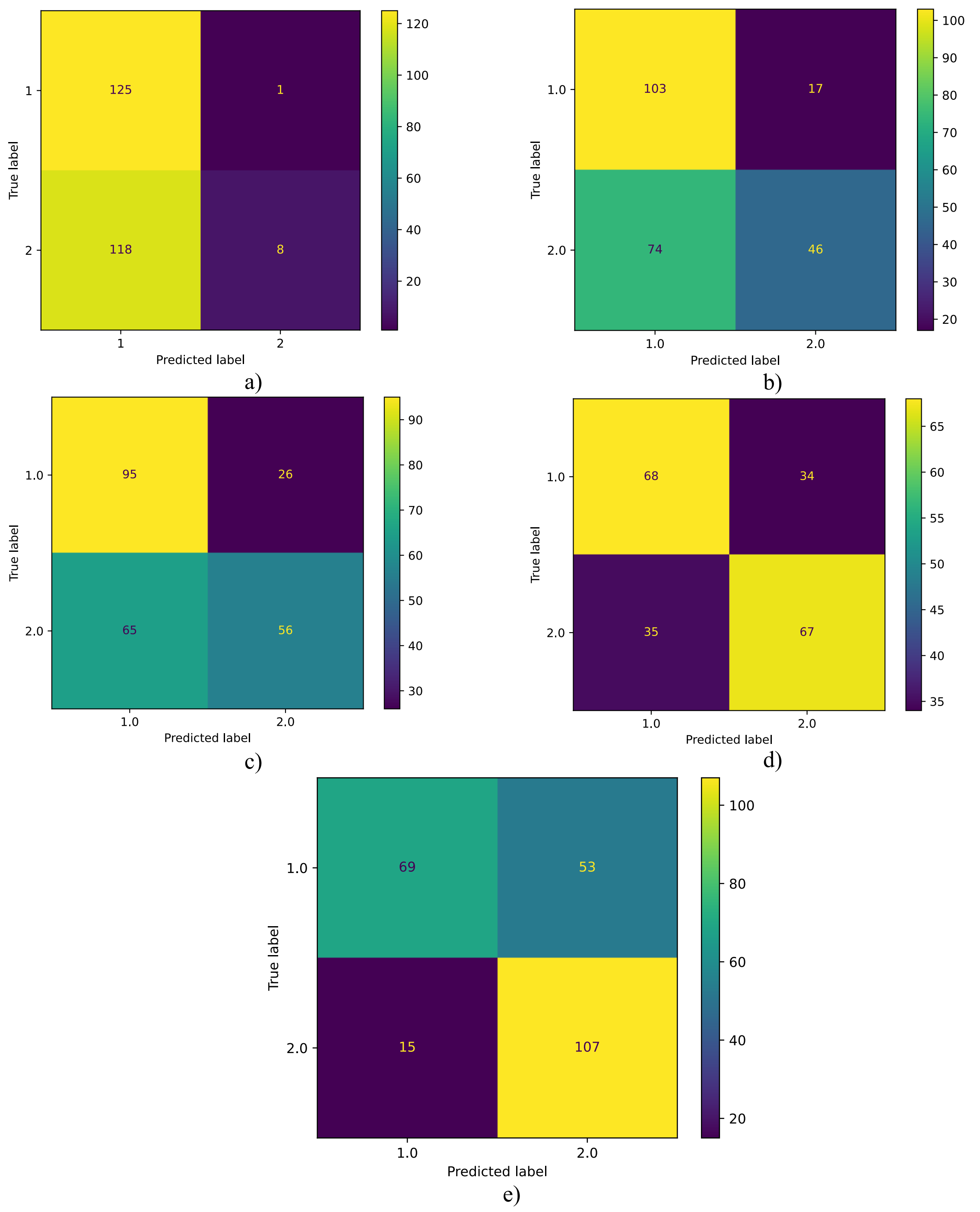

Figure S.3: Confusion matrices for the best performing models across twenty tests derived from the generalisation test for case studies (a) A, (b) B, (c) C, (d) D, and (e) E. Labels ‘1’ and ‘2’ correspond to the ‘Standing’ and ‘Collapsed’ categories, respectively.
